# Effects of Alpha‐Lipoic Acid on Glyphosate‐Treated Human Granulosa Cells: A Cross‐Sectional Study

**DOI:** 10.1002/hsr2.70946

**Published:** 2025-06-25

**Authors:** Sakineh Kaboli Kafshgiri, Armin Attaranzadeh, Hossein Safarpour, Fahimeh Ghasemi, Ebrahim Miri‐Moghaddam

**Affiliations:** ^1^ Royesh Infertility Center Birjand University of Medical Sciences Birjand Iran; ^2^ Milad Infertility Center, Imam Reza Hospital Mashhad University of Medical Sciences Mashhad Iran; ^3^ Cellular and Molecular Research Centre and Department of Medical Biotechnology, Faculty of Medicine Birjand University of Medical Sciences Birjand Iran; ^4^ Department of Molecular Medicine, Faculty of Medicine, Cardiovascular Diseases Research Center Birjand University of Medical Sciences Birjand Iran

**Keywords:** alpha‐lipoic acid, antioxidant, apoptosis, folliculogenesis, glyphosate, infertility

## Abstract

**Backgrounds and Aims:**

An association between Glyphosate (GLY) exposure and a higher frequency of miscarriage and preterm birth has been shown. To evaluate the effects of alpha‐lipoic acid (ALA) on folliculogenesis gene expression and oxidative stress in human GLY‐treated granulosa cells (GCs).

**Methods:**

GCs were obtained from 46 healthy women who had tubal or male infertility factors. The GCs were cultured and treated with GLY (250 μg mL^−1^), ALA (50 μg mL^−^^1^), or 250 GLY + 50 ALA for 24 h. The effect of ALA on apoptosis was evaluated using DAPI staining and flow cytometry.

We assessed the expression of several genes in treated GCs using real‐time PCR. We looked at the Forkhead box protein O1 (*FOXO1*), NADPH oxidase 4 (*NOX4*), Vanin‐1 (*VNN1*), and steroidogenic acute regulatory protein (*StAR*) genes. Additionally, we examined antioxidant genes, including glutathione peroxidase (*GPX*), catalase (*CAT*), and superoxide dismutase (*SOD*).

**Results:**

DNA fragmentation was less pronounced in the ALA‐treated and GLY + ALA groups compared to the GLY‐treated group. ALA treatment reduced the apoptotic ratio in GLY treatment and the combination of GLY + ALA (5.5% and 19.6% vs. 35.8%), respectively. GLY reduced the fold expression of *CAT*, *GPX*, and *SOD* by 0.27, 0.24, and 0.44, respectively. In the ALA treatment group, the fold expression of *SOD*, *CAT*, and *GPX* exceeded that in the untreated group (1.2, 1.02, and 1.7, respectively). GLY decreased the expression of the *VNN1* and *StAR* genes while increasing the expression of the *FOXO1* and *NOX4* genes compared to the control group. Furthermore, ALA decreased *FOXO1* and *NOX4* expression in GCs and increased *VNN1* and *StAR* gene expression.

**Conclusion:**

Exposure to GLY induced apoptosis, and ALA increased the expression of antioxidant genes in GCs. Additionally, ALA can be recommended to reduce the damage caused by oxidants to the female reproductive system.

**Abbreviations**
ALA: alpha‐lipoic acid
CAT: catalase
*FOXO1*
: forkhead box protein O1
GCs: granulosa cells
GLY: glyphosate
GPX: glutathione peroxidase
*NOX4*
: NADPH oxidase 4
SOD: superoxide dismutase
*StAR*
: steroidogenic acute regulatory protein
*VNN1*
: vanin‐1

## 1 Introduction

Infertility remains a widespread problem; 15% of reproductive‐aged couples worldwide are affected by infertility [1]. Among them, one‐third of infertility cases are unexplained cases [2]. Recent studies have shown that certain lifestyles increase the possibility of exposure to endocrine‐disrupting chemicals that may cause disorders of the reproductive system [3, 4]. Epidemiological studies have shown a close relationship between environmental and occupational exposure to pesticides and unpleasant disorders of the reproductive systems of men and women [5]. These adverse effects may occur by increasing the risk of infertility, abortion, stillbirth, low birth weight, or congenital anomalies [2, 6].

Currently, agriculture is highly dependent on herbicides and pesticides. Glyphosate (GLY) or *N*‐(phosphonomethyl) glycine is considered to be among the most important herbicides used in modern agriculture [7, 8]. The primary mechanism of action for GLY is its ability to block the shikimate acid pathway. In plants, fungi, and some microorganisms, this pathway is essential for synthesizing aromatic amino acids (such as phenylalanine, tyrosine, and tryptophan). GLY inhibits the enzyme 5‐enolpyruvylshikimate‐3‐phosphate synthase (EPSPS), which is a critical step in the shikimate pathway. By inhibiting EPSPS, GLY prevents the production of aromatic amino acids. These amino acids are crucial for protein synthesis, growth, and various cellular processes [9]. Studies have shown that the side effects of GLY are associated with various diseases, such as cancer, metabolic and endocrine disorders, imbalanced intestinal flora, autism spectrum disorders, and infertility [7, 9, 10, 11]. While experimental evidence consistently shows GLY toxicity in reproduction and development, human studies are scarce and sometimes controversial. GLY exposure has implications not only for mothers and fetuses but also for offspring across several generations. In vitro models have also shown that GLY affects reproductive cells and related metabolic pathways [12]. Granulosa cells (GCs) play a crucial role in maintaining two‐way communication with oocytes, and their impact on regulating the growth of oocytes varies depending on their level of maturity and the nature of their communication [13, 14]. Alterations such as decreased proliferation, cell calcification, and atrophy have been observed in ovarian cells exposed to GLY [11]. Vanin‐1 (*VNN1)*, Forkhead O (*FOXO*), NADPH oxidase 4 (*NOX4)*, and steroidogenic acute regulatory protein (*StAR*) are involved in the regulation of wide‐ranging cellular processes related to the female reproductive system [15, 16, 17].

Several studies have focused on the role of antioxidant supplementation in improving oocyte and sperm performance to improve the rate of spontaneous pregnancy or during in vitro fertilization in infertile patients. Alpha‐lipoic acid (ALA) is well known for its antioxidant effects on numerous biological procedures and for scavenging ROS [18]. Research has shown that ALA promotes follicular growth, oocyte maturation, and embryo development [19, 20].

The effect of pesticides on oocyte maturation in animal models showed that females were more likely to be toxified than males were [21]. Little is known about the effects of this herbicide on human GCs. The present study aims to evaluate the effects of GLY on the expression of folliculogenic genes. In addition, due to the role of GLY in the occurrence of oxidative stress and the use of ALA as an antioxidant agent, we investigated the protective effect of ALA against OS caused by GLY in human GCs.

## 2 Methods

### 2.1 Study Population

In this study, luteal GCs were isolated from the follicular fluid of 46 females aged less than 40 years. In this age range, some biological processes such as cellular repair mechanisms and metabolic flexibility may be stronger, and by choosing younger people, the potential for early intervention and long‐term health benefits can be evaluated. This study protocol was reviewed and approved by the Ethics Committee of Birjand University of Medical Sciences (IR.BUMS.REC.1398.393). Informed consent was obtained from all individual participants included in the study. The patients received intracytoplasmic sperm injections or IVF treatment for the first time. The study's inclusion criteria were as follows: spouses with a total sperm count of 1 × 10^6 ^mL^−1^, a sperm motility rate of < 5%, an abnormality index of > 95% based on the WHO guidelines [22], and females with tubular factor infertility. Patients with human immunodeficiency viruses, hepatitis C and B viruses, polycystic ovarian syndrome, or cytomegalovirus infectivity were excluded from the study. Cumulus‐oocyte complexes (COCs) were collected from healthy women who underwent IVF or intracytoplasmic sperm injection (ICSI). Cumulus oocyte complexes were collected from the ovary by an expert gynecologist using a transvaginal ultrasound‐guided suction system.

### 2.2 Human GC Collection and Culture

GCs were isolated from the COCs using a fine sterile pipette. The isolated specimens were placed inside preheated medium containing hyaluronidase (Sigma Aldrich, Germany) at 37°C. Then, the GCs were incubated in an enzymatic solution containing hyaluronidase for 3 h and rinsed in triplicate with phosphate‐buffered saline (PBS). The cells were cultured in DMEM/F12 containing 10% fetal bovine serum, 1% penicillin, and 1% streptomycin, followed by incubation at 37°C with 5% CO_2_ for 5 days. To assess the effect of GLY and ALA on GCs, they were treated with various concentrations of GLY (Bayer, Germany) (250 μg mL^−1^), ALA (Webber Naturals, USA: 20340) (50 μg mL^−1^), or their mixture (250 GLY + 50 ALA) for 24 h.

### 2.3 Assays of Morphological Changes in GCS Treated With GLY and ALA by DAPI Staining

Morphological changes in the nuclei of treated GCs were evaluated with 4′,6′‐diamidino‐2‐phenylindole (DAPI) staining. The GCs were treated with different concentrations of GLY and ALA, washed with PBS, and fixed with methanol at room temperature. Following fixation, the cells were stained with 0.5 μg mL^−1^ DAPI for 5 min, and the nuclei were observed using a fluorescence microscope.

### 2.4 Evaluation of Apoptosis in GCS Treated With GLY or ALA by Flow Cytometry

Evaluation of apoptosis in GCs treated with GLY, ALA, or their combination (GLY + ALA) was performed using an Annexin V‐FITC/PI kit and flow cytometry. The cells were washed with PBS, trypsinized, and resuspended in 500 µL of 1× binding buffer and 5 µL of Annexin V‐FITC. Then, 5 µL of propidium iodide (PI) was added, and the mixture was incubated for 30 min in the dark. Cell analysis by flow cytometry was performed using WinMDI software.

### 2.5 Evaluation of Antioxidant and Folliculogenesis Gene Expression by Real‐Time PCR

The mRNA expression levels of antioxidant *(SOD*, *GPX*, and *CAT*) and folliculogenesis (*FoxO1, NOX4*, *StAR*, and *VNN1*) genes were assessed in GCs treated with GLY, ALA, or their combination by real‐time PCR. Total RNA was extracted from GCs using a high‐purity RNA isolation kit (Denazist, Iran) according to the manufacturer's instructions. The quality and quantity of the isolated mRNA were assessed, and first‐strand cDNA synthesis was performed using a reverse transcription kit (Thermo Scientific, USA) according to the manufacturer's instructions. PCR was run on a 20 μL mixture containing 10 μL of SYBR Green master mix (Pars Tous, Iran), 1 μL of cDNA, 7 μL of nuclease‐free water, and 1 μL of each primer using a real‐time PCR system (Bio‐Rad CFX96). The reactions were carried out in triplicate for cDNA obtained from each culture. The primer sequences (Sina Clon, Tehran, and IRAN) used are listed in Table 1. The expression levels were normalized to those of glyceraldehyde 3‐phosphate dehydrogenase, and the relative expression was quantified according to the comparative CT method.

**Table 1:** Applied primer sequence for the detection of gene expression by real‐time PCR.

Gene	Forward 5ʹ → 3ʹ	Reverse 5ʹ → 3ʹ	Annealing (°C)
*SOD*	AGGGCATCATCAATTTCGAGCA	AATAGACACATCGGCCACACC	58
*GPX*	AAGTGTTGCTGAGTGAGG	TGTGCTATGAAGTCTGAGG	58
*CAT*	ATGGCTCATTTTGACCGAGA	ATTTCACTGCAAACCCACGAG	58
*FOXO1*	TCACCCAGCCCAAACTACCAA	CTTCAAGAGTCCAGGCGCACA	58
*Vnn1*	CTATGCCAGCAGTATAGAAGC	CGCAAGTGTTTAAATTAGTCG	58
*NOX4*	CAGTCACCATCATTTCGGTCA	GATGAACCCCAAATGTTGCTT	58
*STAR*	CCACAGACTTCGGGAACATGCC	CCACCCCTTGAGGTCGATGC	58
*GAPH*	AAGCTCATTTCCTGGTATGACAACG	TCTTCCTCTTGTGCTCTTGCTGG	59

### 2.6 Statistical Analysis

The collected data were analyzed using one‐way ANOVA and Tukey's test in the GraphPad Prism software package (version 9). The data are reported as the mean ± standard deviation, and *p* < 0.05 was considered to indicate statistical significance.

## 3 Results

**Figure 1:**
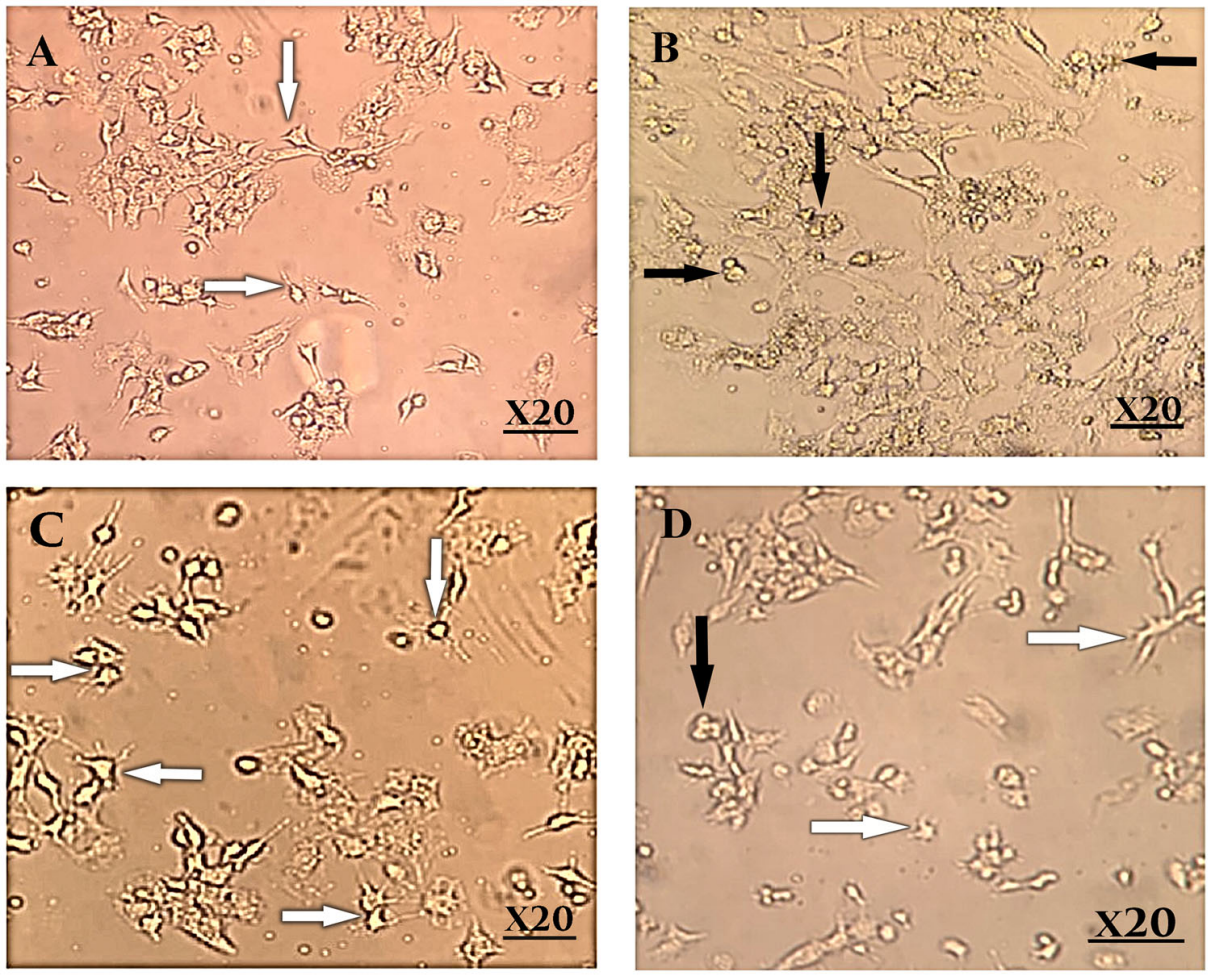
Fluorescence micrographs of GCs treated with various concentrations of glyphosate, alpha‐lipoic acid, and synergetic combinations of glyphosate + alpha‐lipoic acid. (A) Control group; (B) glyphosate (250, μg mL^−1^); (C) alpha‐lipoic acid (50 μg mL^−1^); (D) synergetic combination (ALA + GLY) after 24 h of treatment with DAPI (20× magnification).

**Figure 2:**
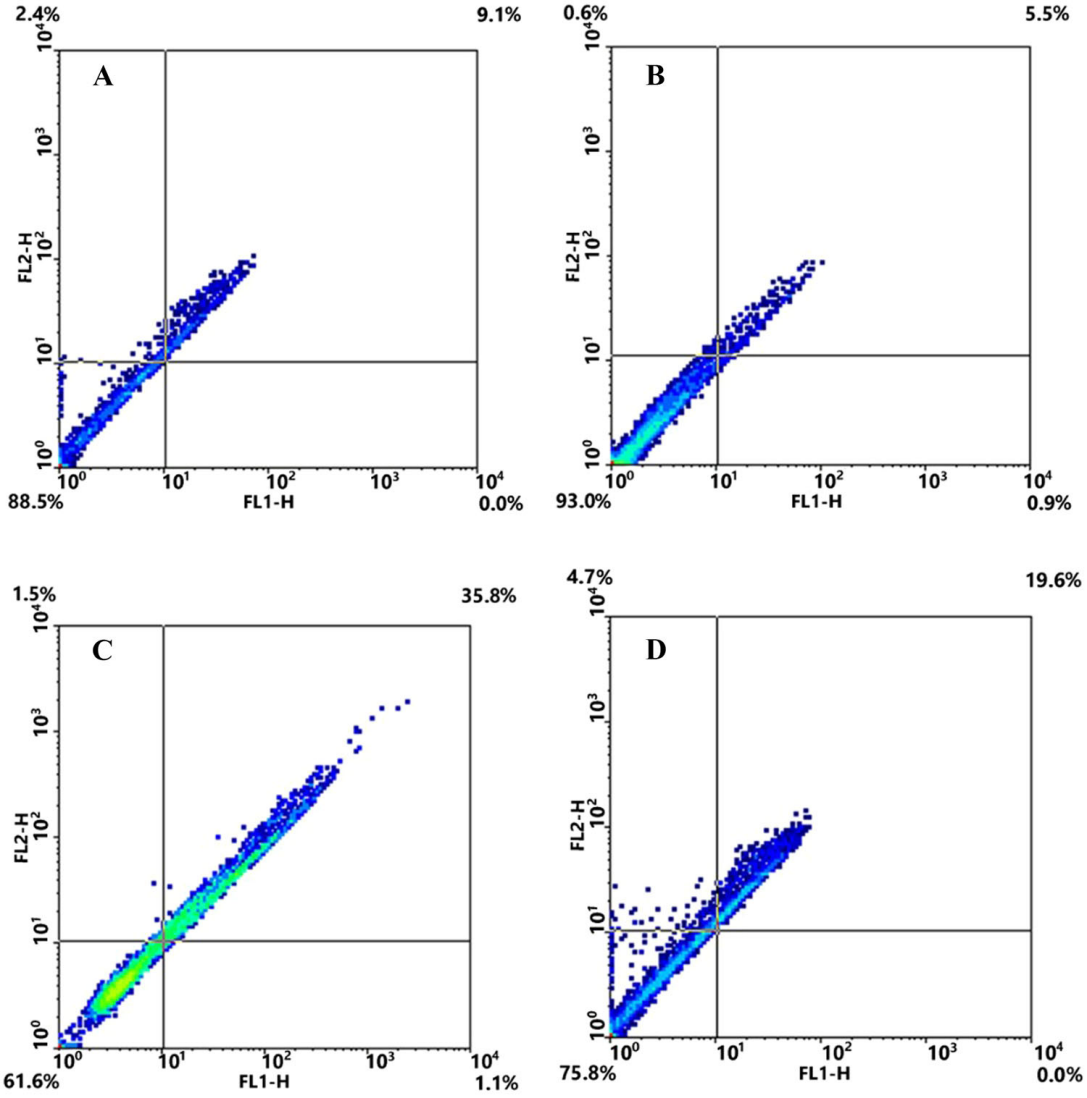
Flow cytometry results (GC apoptosis after treatment with different concentrations of alpha lipoic acid, glyphosate, and the synergetic combination (ALA + GLY) for 24 h) based on the Annexin‐v/PI assay. (A) Control (untreated); (B) alpha lipoic acid (50 µg/mL); (C) glyphosate (250 µg/mL); (D) ALA + GLY (µg/mL).

In the exposed GLY and GLY + ALA groups, the GCs exhibited cell shrinkage, a reduced cell volume, and plasma membrane distortion, whereas these changes were not observed in the exposed ALA group. DAPI staining revealed DNA fragmentation in the GLY‐treated groups at 250 μg/mL compared to the untreated group. In contrast, DNA fragmentation did not occur in the ALA‐treated groups (50 μg mL^−1^) or in the GLY + ALA (250 GLY + 50 ALA μg mL^−1^) group compared to the GCs of the GLY‐treated groups. DAPI staining resulted in completely round nuclei in the untreated cells, whereas apoptotic features such as condensed chromatin and fragmented nuclei were observed in the GLY‐treated cells (Figure 1). The results indicated that GLY led to a greater apoptotic ratio in GLY‐treated cells than in untreated cells (35.8% vs. 9.1%). On the other hand, compared with no treatment, ALA treatment reduced the apoptotic ratio in GLY‐treated cells (5.5% vs. 35.8%), while combined treatment with GLY (250 μg mL^−1^ + 50 ALA μg mL^−1^) led to a reduction in the apoptotic ratio compared to that in GLY‐treated cells (19.6% vs. 35.8%) (Figure 2).

**Figure 3:**
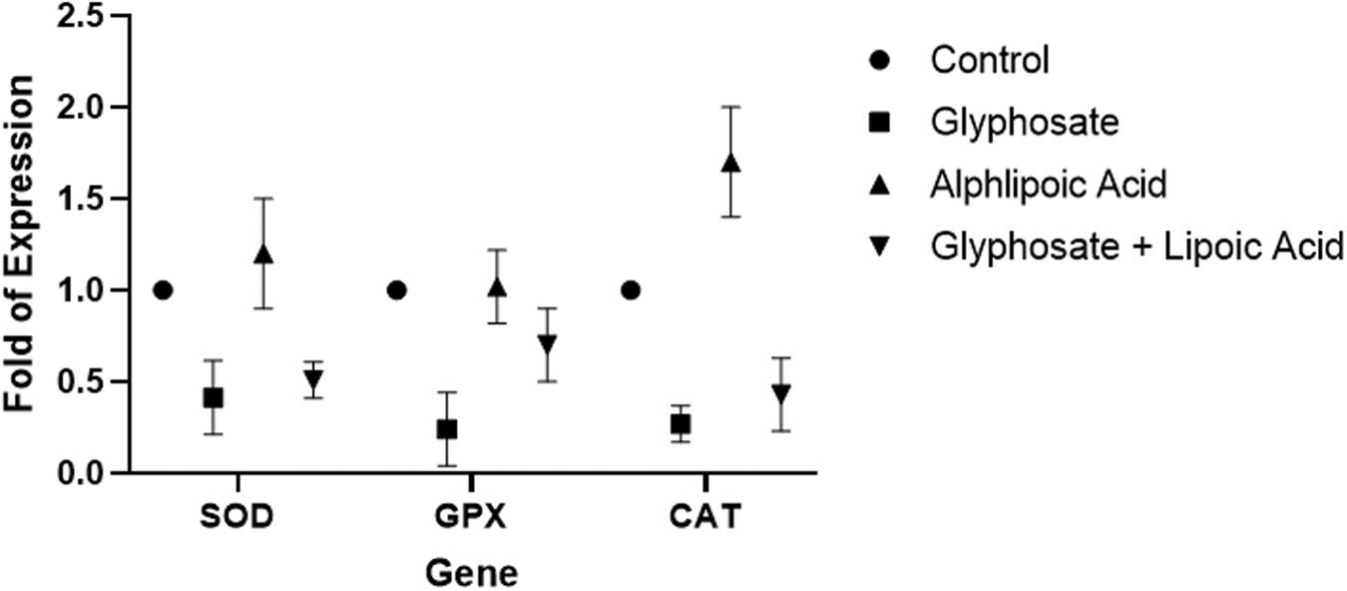
Real‐time PCR analysis of representative genes and the gene expression of *SOD*, *GPX*, and *CAT* in GCs treated with GLP, ALA, or the synergetic combination of glyphosate + alpha‐lipoic acid. The data are expressed as the mean and standard deviation (SD); values are significantly different (*p* < 0.05) from those of the controls.

The results revealed that in the GLY‐treated group, the fold of expression of *SOD*, *GPX*, and *CAT*, compared to the control group were 0.44, 0.24, and 0.27, respectively). In the ALA treatment group, the fold expression of *SOD*, *CAT*, and *GPX* exceeded that in the untreated group (with fold changes of 1.2, 1.02, and 1.7, respectively). In the combination treatment of GLY + ALA, there was a reduction in the fold expression of *SOD*, *GPX*, and *CAT* compared to untreated cells (with fold changes of 0.51, 0.7, and 0.43, respectively) (Figure 3).

**Figure 4:**
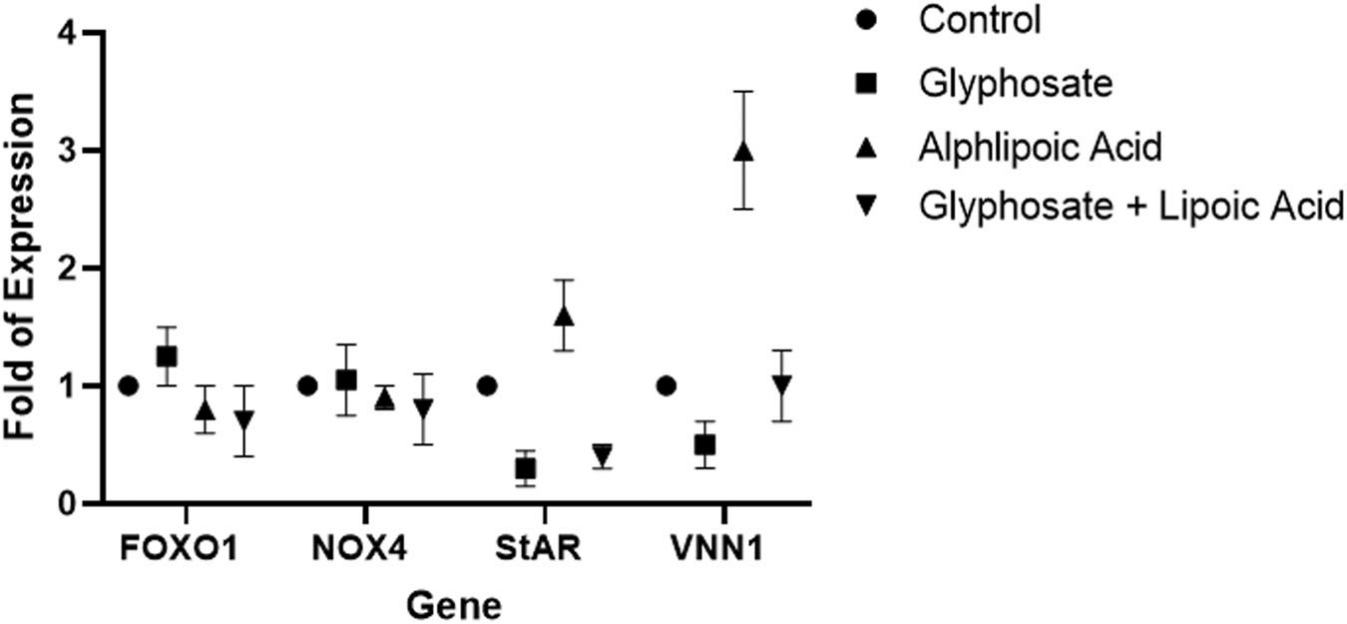
Real‐time PCR analysis of the expression of the representative genes *FOXO1*, *NOX4*, *VNN1*, and *StAR* in GCs treated with glyphosate, alpha‐lipoic acid, or combinations of glyphosate + alpha‐lipoic acid in GCs. The data are expressed as the mean ± SD. The values are significantly different (*p < 0.05) (**p < 0.01) from those of the controls.

The results demonstrated that GLY elevated the transcription of *FOXO1* and *NOX4* compared to the control group, not significant (*p* > 0.05), while the expression of *VNN1* and *StAR* in GCs showed significantly reduction (*p* < 0.01 and *p* < 0.001, respectively*)*. In the ALA‐treated group, there was no significant difference in the expression of *Foxo1* and *Nox4* (*p* > 0.05). However, the expression of *StAR* and *VNN1* increased significantly (*p* < 0.01 and *p* < 0.01, respectively). In the combination (GLY + ALA) treated group, *FOXO1* and *NOX4* expression decreased (*p* > 0.05), while *VNN1* expression increased not significant (*p* > 0.05). Notably, *StAR* expression showed a significant decrease (*p *< 0.01) (showed in Figure 4).

## 4 Discussion

GLY is a widely used herbicide, and its impact on the environment and human health has been a topic of debate and research for many years. This study showed that GLY induced toxic and necrotic effects on GCs, while treatment with ALA reduced these characteristics and morphological changes. These features and morphological changes in the cells likely indicated GC apoptosis. Lu et al. demonstrated that GLY causes cellular senescence, promotes senescence‐associated β‐galactosidase activity, and enhances p53, p21, and p16 protein expression in human aortic vascular smooth muscle cells [23]. Exposure to GLY and its byproduct led to a decrease in the production of two genes, *TUBB3* and *GAP43*, which play a role in creating proteins for nervous system structure and the growth of axons [24].

This study showed that GLY exacerbated the process of apoptosis in human GCs, while ALA supplementation improved the antiapoptotic effects of GLY‐induced stress. Apoptosis in GCs may be caused by multiple parameters and via several pathways. Martini et al. reported that, compared with a control, GLY increased the number of apoptotic fibroblasts by 11% [25]. Zhao et al. reported that GLY inhibits cell proliferation and induces apoptosis in Sertoli cells [26]. Wang et al. reported that exposure to pesticides disrupts the growth of GCs via oxidative stress, apoptosis, and autophagy [27]. Xing et al. demonstrated that GLY disrupts the functions of mitochondria, the endoplasmic reticulum, the lysosome, the Golgi apparatus, and ribosomes in porcine oocytes, potentially resulting in reproductive harm to their developing embryo and offspring [28]. The malathion‐exposed GCs had significantly higher mRNA levels of *BCL2*, *BAX*, *BCL2*/*BAX*, *BAK*, *CASP9*, and *CASP3*, which indicates that malathion induces mitochondrial damage upstream of these genes, thereby activating *CASP9* and *CASP3* to induce apoptosis [29].

The results showed that GLY decreased the transcription of the *SOD*, *GPX*, and *CAT* genes. However, in the samples treated with ALA, the expression of these genes increased. In the female mammalian ovary, apoptosis and protein oxidation increase during follicular atresia, and antioxidant levels decrease in the follicles [30]. Cells protect themselves against ROS through intracellular glutathione proteins and antioxidant enzymes. Additionally, CAT and GPX, which are involved in the glutathione cycle, convert H_2_O_2_ to water and oxygen [31]. They observed a significant increase in *SOD2* mRNA levels, which is considered the first‐line defense mechanism against oxidative damage; this change could be a compensatory mechanism in response to increased ROS generation. A similar increase was observed for the other enzymes with protective effects (e.g., GPX1, PRDX2, and CAT) due to the oxidative damage that is involved in the regulation of redox signaling. The possible mechanism of ROS production and toxicity induction in the reproductive system by insecticides leads to follicle destruction by reducing antioxidant SOD activity [32, 33]. The release of enough ROS around mitochondria can trigger the activation of redox‐sensitive enzymes that play a role in safeguarding signaling processes. These pathways work to diminish the impact of ischemic harm on both the mitochondria and cells within that specific region [34].

This study demonstrated that the effect of GLY on GCs led to a decrease in *VNN1* and *StAR* gene expression and an increase in *FOXO1* and *NOX4*. *VNN* belongs to a protein family that has a minimum of three members [35]. The expression of the *VNN1* gene increases over time and with the size of the follicle. This finding suggested that this gene could serve as a potential marker for follicular growth and/or differentiation [36]. Additionally, *VNN1* controls the reaction of tissues to oxidative stress by adjusting the GSH reserves in mice [37]. In this regard, Girard et al. reported the oxidative stress response of *VNN1*, which in turn led to decreased expression of the gene involved in the proliferation of cells; the subsequent disruption was related to oxidative stress and GC atresia [38]. This study showed that *VNN1* expression decreased with increasing GLY concentration, indicating that atresia is a sign of follicle integrity.

StAR is the most important steroid enzyme in GCs and is involved in the transfer of cholesterol from the outer to the inner mitochondrial membrane and its conversion to pregnenolone. Ovarian steroid regulatory genes keep follicle steroid hormones stable, contribute to follicle growth, and maintain follicle and luteal function [39]. Alterations in steroid enzyme expression and hormone secretion in GCs are the main causes of impaired follicular development and maturation, leading to the premature cessation of follicle growth in polycystic ovarian cysts [40]. The present study revealed that the expression of *StAR* decreased significantly in the GLY treatment group, while the decrease in the group treated with ALA was very low, and the decrease in the synergistic group was greater than that in the group treated with ALA. These negative effects were also consistent with the higher levels of oxidative stress and morphological changes, and showed that external stimuli can have destructive effects on ovarian cells. Moreover, antioxidants improve steroidogenesis by increasing their expression.

The NOX4 isoform is expressed in human GCs and produces ROS in the ovary, which may be related to female fertility factors. Egg maturation is regulated by NOX‐derived ROS, which are second messenger molecules in the protein kinase C signaling pathway [16, 41]. Our results showed that *NOX4* expression was not significantly increased in GCs exposed to GLY but was decreased in the ALA and ALA + GLY treatment groups. Tousson et al. showed that treatment with different concentrations of grape extract reduced the ROS content and *NOX4* mRNA expression, while the opposite was observed in human GCs. Since NOX4 is the main NOX enzyme expressed in GCs, ROS content can be regulated by antioxidants in human GCs, possibly through the transcriptional effect of *NOX4* [42].

FOXO proteins are transcription factors that contribute to the regulation of a wide range of cell functions. The role of FOXO has been highlighted in mammalian ovaries. Kajihara et al. showed that FoxO1 in the endometrium is crucial for the endometrial transformation that occurs during menstruation as well as for protecting pregnant women from oxidative stress [43]. FOXOs play an active role in encouraging cell death through both mitochondrial and nonmitochondrial means. These proteins stimulate the production of death receptor ligands, such as Fas ligands and tumor necrosis factor‐related apoptosis‐inducing ligands, as well as various Bcl‐2 family members, such as Bim, bNIP3, and Bcl‐XL [44]. This study showed that *FOXO1* expression increased in GCs that were exposed to GLY and decreased in those in the ALA and ALA + GLY groups. The increased expression of *FoxO1* in the GCs of oxidant‐treated mice was consistent with the increase in apoptotic signals [45]. The results of this study were consistent with the results of a study carried out by Shen et al.

ALA is an antioxidant that directly scavenges free radicals, and it can also preserve the innate cellular antioxidant defense system [46]. Furthermore, the direct/indirect scavenging activity of ALA against free radicals occurs through the retention of antioxidants such as GSH recycling and cystathionine β‐synthase recycling [47]. ALA is involved in inhibiting advanced glycation end products generated through ROS production. In addition, ALA has been reported to prevent the apoptosis of human umbilical vein endothelial cells [48]. ALA increases the follicle growth rate by affecting the expression of follicle growth regulatory genes in bovine secondary preantral follicles (e.g., *FSHR*, *LH/CGR*, *IGF‐1*, *BMPR1a*, *TGFβR1*, *TGFβ1*, *ActRIIB*, *GDF9*, and Activin A) [49]. ALA may protect human reproductive cells by maintaining cell viability and modulating proliferation pathways. ALA might influence steroid hormone synthesis within the testes and ovaries. GLY inhibits the shikimate pathway in plants. However, since this pathway doesn't exist in animal cells, its influence on related pathways may still protect cells. The results of the present study showed that ALA reduced the destructive effect of GLY on GCs and had antioxidant effects.

In this study, we encountered several limitations. Firstly, we did not explore the effects of GLY across a wider range of doses and time points. Additionally, we did not evalutate its impact on other cell types or utilized animal models. Looking ahead, we recommend conducting proteomic and metabolic analyses in future study to gain a deeper understanding of the underlying mechanisms.

## 5 Conclusion

The results showed that exposure to GLY causes morphological changes in the structure of GCs, which may interfere with reproductive system function. Moreover, GLY reduced *VNN1* and *StAR* expression and increased *FOXO1* and *NOX4* expression. However, treatment with ALA antioxidants increased the expression of *VNN1* and *StAR* and reduced the expression of the *FOXO1* and *NOX4* genes. Finally, GLY decreased the transcription of the *SOD*, *GPX*, and *CAT* genes, but ALA increased the expression of these genes. ALA could be a reliable antioxidant due to minimal damage to the female reproductive system. Although several studies have addressed the effect of ALA on follicle maturation, little is known about the effect of ALA on the expression of follicle‐regulating genes and apoptosis‐inducing genes; therefore, further studies are needed.

## Author Contributions

**Sakineh Kaboli Kafshgiri:** conceptualization, methodology, writing – original draft, and project administration. **Armin Attaranzadeh:** conceptualization, writing – original draft, and supervision. **Hossein Safarpour:** writing – original draft, methodology, and data curation. **Fahimeh Ghasemi:** writing – original draft and methodology. **Ebrahim Miri‐Moghaddam:** conceptualization, writing – review and editing, methodology, and supervision.

## Ethics Statement

This study protocol was reviewed and approved by the Ethics Committee of Birjand University of Medical Sciences (IR.BUMS.REC.1398.393). Informed consent was obtained from all individual participants included in the study.

## Conflicts of Interest

The authors declare no conflicts of interest.

## Transparency Statement

The corresponding author, Ebrahim Miri‐Moghaddam, affirms that this manuscript is an honest, accurate, and transparent account of the study being reported; that no important aspects of the study have been omitted; and that any discrepancies from the study as planned (and, if relevant, registered) have been explained.

## Data Availability

The authors confirm that all data generated or analyzed and the data supporting the findings of this study are available within the article.
